# Prefrontal Hemodynamic Changes Associated with Subjective Sense of Occlusal Discomfort

**DOI:** 10.1155/2015/395705

**Published:** 2015-05-18

**Authors:** Yumie Ono, Goh Kobayashi, Rika Hayama, Ryuhei Ikuta, Minoru Onozouka, Hiroyuki Wake, Atsushi Shimada, Tomoaki Shibuya, Katsushi Tamaki

**Affiliations:** ^1^Health Science and Medical Engineering Laboratory, Department of Electronics and Bioinformatics, School of Science and Technology, Meiji University, Room A806, 1-1-1 Higashi-Mita, Tama-ku, Kawasaki, Kanagawa 214-8571, Japan; ^2^Department of Prosthodontic Dentistry for Function of TMJ and Occlusion, Kanagawa Dental University, 82 Inaoka-cho, Yokosuka, Kanagawa 238-8869, Japan; ^3^Department of Judo Therapy and Medical Science, Faculty of Medical Science, Nippon Sport Science University, 1221-1 Kamoshida-cho, Aoba-ku, Yokohama 227-0033, Japan

## Abstract

We used functional near-infrared spectroscopy to measure prefrontal brain activity accompanying the physical sensation of oral discomfort that arose when healthy young-adult volunteers performed a grinding motion with mild occlusal elevation (96 *μ*m). We simultaneously evaluated various forms of occlusal discomfort using the visual analogue scale (VAS) and hemodynamic responses to identify the specific prefrontal activity that occurs with increased occlusal discomfort. The Oxy-Hb responses of selected channels in the bilateral frontopolar and dorsolateral prefrontal cortices increased in participants who reported increased severity of occlusal discomfort, while they decreased in those who reported no change or decreased occlusal discomfort during grinding. Moreover, the cumulative values of Oxy-Hb response in some of these channels were statistically significant predictive factors for the VAS scores. A generalized linear model analysis of Oxy-Hb signals in a group of participants who reported increased discomfort further indicated significant cerebral activation in the right frontopolar and dorsolateral prefrontal cortices that overlapped with the results of correlation analyses. Our results suggest that the increased hemodynamic responses in the prefrontal area reflect the top-down control of attention and/or self-regulation against the uncomfortable somatosensory input, which could be a possible marker to detect the subjective sense of occlusal discomfort.

## 1. Introduction

Dental outpatients often present with symptoms related to uncomfortable sensations such as pain or discomfort in the oromandibular area. Although objective findings that match the symptoms can be easily confirmed in the majority of cases, the evaluation of patients with vague complaints in the intraoral area such as occlusal discomfort without any identifiable organic cause (occlusal dysesthesia) has been a growing problem in recent years [1]. That dental therapy such as occlusal adjustment mostly does not relieve any discomfort in these patients raises the hypothesis that they develop a perception bias or a modified sensory processing network in their body image of the orofacial area. Because many cases of occlusal dysesthesia accompany psychiatric disorders such as somatoform disorders [1], it appears necessary to clarify how the emotional response toward occlusal discomfort is processed and represented in the higher cognitive areas of the brain. If the subjective symptom of occlusal discomfort can be quantified from the regional brain activity of the patient, the clinician can diagnose the occlusal dysesthesia based on the mismatch of the existing occlusal discomfort represented in the brain activity and the lack of objective findings in the oral area. This approach will enable clinicians to prevent unnecessary dental treatment to patients with occlusal dysesthesia and to allow patients to understand the actual disease behind their occlusal problem. Thus, patients will be able to undergo appropriate treatment, which will improve their symptoms and quality of life.

We therefore studied regional brain activity corresponding to experimentally generated occlusal discomfort in participants without stomatognathic symptoms and determined the cortical areas in which the emotional response to modified and uncomfortable oral sensations is processed. Previous neuroimaging studies on occlusal discomfort have focused on comparing the basal brain activity between patients and control participants in a resting state [2, 3]. Although these studies are useful for extracting the basal brain activity specific to patients, it is hard to determine whether the extracted neuronal changes reflect the perceived discomfort* per se* or other “secondary” neuronal activity caused by their symptoms, such as stress responses and altered mood. We therefore used an “active” grinding paradigm in participants without stomatognathic symptoms to extract the regional brain activity that specifically responds to the elevation of perceived discomfort in participants. Considering that the ultimate goal of this study is to develop a monitoring system for occlusal discomfort in the clinical setting, we selected the prefrontal cortex, a top-down control center for somatosensory perception [3–5], as a region of interest for its accessibility.

We used functional near-infrared spectroscopy (fNIRS) to monitor prefrontal responses over other traditional brain imaging techniques for three reasons: first, fNIRS allows participants to take natural occlusal positions in a sitting position while undergoing a scan. Second, fNIRS has relatively better tolerance to body movement, given that the optodes are tightly attached to the head and that the head motion is restricted [6]. Third, the recent development of portable fNIRS systems makes it possible to continuously monitor hemodynamic responses in a real-world environment [7]. We also intended to reject pseudohemodynamic responses from changes in cutaneous and muscle blood flow during grinding motions, as well as motion artifacts from jaw movements, by subtracting hemodynamic signals during grinding with elevated occlusion from those during grinding with natural occlusion. To ensure that the subtraction could cancel out artifacts from muscle-related hemodynamic signals, the strength of masseter muscle activity during grinding motion with and without occlusal elevation was compared in a small group of participants. We gave a fixed height of occlusal elevation that evoked various intensities of discomfort among participants with individual occlusion to explore the spectrum of interactions of perceived discomfort on neural substrate activity.

## 2. Materials and Methods

### 2.1. Participants

Twenty-five participants aged from 21 to 49 years took part in the fNIRS experiment (14 males, 11 females, 28.9 ± 1.0 years) and six participants aged from 25 to 34 years took part in the electromyography (EMG) experiment (three males, three females, 29.5 ± 1.6 years). All were free of medical and psychiatric symptoms and had no perceived symptoms in their stomatognathic system. A clinician (GK or RH) interviewed all participants and assessed their stomatognathic function upon arrival to the lab and ensured that their individual occlusion was within the normal range. Written informed consent was obtained from each participant after a full explanation of the experiment was provided. The study followed the protocol for the use of human participants and was approved by the Ethics Committee of the Kanagawa Dental University Hospital.

### 2.2. Task Paradigm

**Figure 1:**
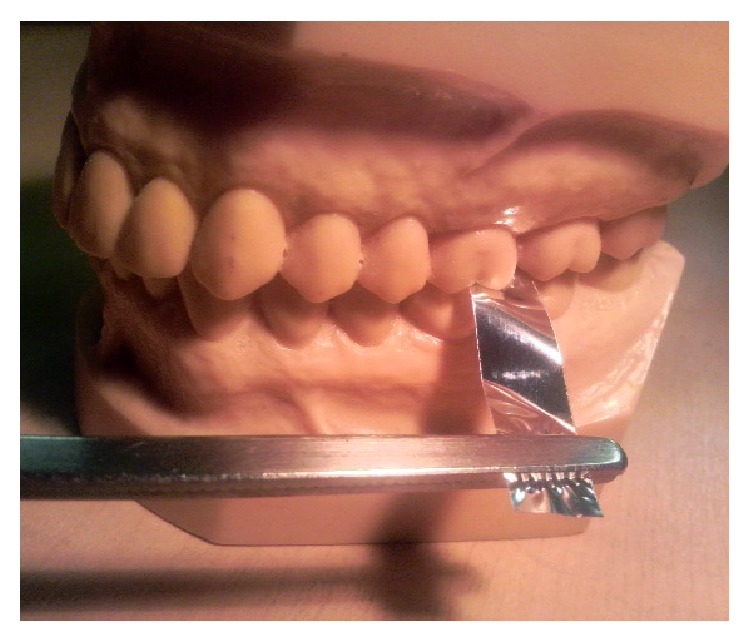
Simulation of occlusal discomfort using active grinding paradigm.

Occlusal discomfort was simulated by grinding the teeth with stacked, tasteless, and odorless, metal strips (eight 12 *μ*m strips stacked to 96 *μ*m of thickness; Artus, Englewood, NJ) placed on the occlusal surface of the first molar of the habitual chewing side (Figure 1). The thickness of the metal strip was selected to be enough to cause obvious discomfort during grinding in most of the participants, but cause neither injury of the periodontal tissue nor tooth movement [8–10].

### 2.3. Data Acquisition

A single session of fNIRS measurement was performed using a block design, which consisted of alternating 30 s of grinding and 40 s of rest, repeated five times. We used the 22-channel fNIRS topography system ETG-7100 (Hitachi Medical Corporation, Tokyo, Japan), arranged into a 3 × 5 optical probe array positioned over the bilateral prefrontal cortices. The lowest optode row was aligned with the line connecting F7-F8 in the international 10–20 system. Interoptode distance was 3 cm for each source detector pair. Using a 3D digitizer (PATRIOT; Polhemus, Colchester, VT), the coordinates of all probe positions and the anatomical landmark positions (nasion, inion, auricles, and Cz) of each participant were obtained immediately after data collection.

Participants performed two sessions of fNIRS measurement in which they grinded their teeth with or without metal strips on their first molar. The experimenter pinched the tip of the metal strips using locking tweezers and applied them to the surface of the lower molar of a participant 10 s before each trial. Participants kept the metal strips in their mouth in the intercuspal position after they were applied to the tooth. Verbal instructions given at the beginning and the end of the grinding period told the participants when to start and stop grinding, respectively. The metal strips were taken out of the mouth immediately after the grinding period each time. In trials without metal strips, the experimenter inserted only the tweezers into participants' mouths in the same way as in the trials with strips. The order of the two sessions was randomly assigned, and participants were asked to grind their teeth mildly and uniformly across trials and sessions. To ensure that participants could perform grinding with comparable muscle strength between sessions, another group of six participants who reported increased discomfort while grinding with strips compared with grinding without strips performed the same experimental tasks while EMG was recorded from the masseter (WEB-1000; Nihon Koden, Tokyo, Japan) on the grinding side without fNIRS recording.

At the end of each session, all participants evaluated the subjective severity of discomfort using a visual analog scale (VAS). The VAS varied from 0 (a state with no discomfort at all) to 100 (a state of intolerable discomfort). “Discomfort” was defined using the following six criteria: (i) the feeling of a “too-high” bite; (ii) jitteriness of the tongue, tooth, and oral cavity; (iii) difficulty in chewing; (iv) unstable intercuspal position (IP); (v) uneven contact of bilateral molar teeth; and (vi) tooth pain [11], each of which the participants were asked to evaluate. We subtracted VAS scores in the session when participants ground with metal strips from that without metal strips for each of these six criteria and used them as an index of subjective discomfort with elevated occlusion (hereinafter referred to as dVAS; ranges from −200 to 200) for further analysis.

### 2.4. Data Analysis

Changes in cerebral Oxy-Hb responses were averaged over five trials using a built-in function of the fNIRS system (ETG-7100 V3.13 K; Pre: 5.0 s, Recovery: 20.0 s, Post: 5.0 s). We used in-house developed software running on Matlab (Mathworks, Natick, MA, USA) for signal processing. To cancel out artifacts in Oxy-Hb responses related to jaw movement and muscle-originating hemodynamic responses, we subtracted Oxy-Hb responses during grinding with metal strips from those without metal strips (ΔOxy-Hb). Baseline correction was applied to the ΔOxy-Hb responses so that the value at the task onset was set to zero. The amplitude of the hemodynamic signal was further normalized by dividing the ΔOxy-Hb values by the standard deviation of those during the 5 s before task onset. These baseline-corrected and normalized ΔOxy-Hb responses were used for further analysis.

**Figure 2:**
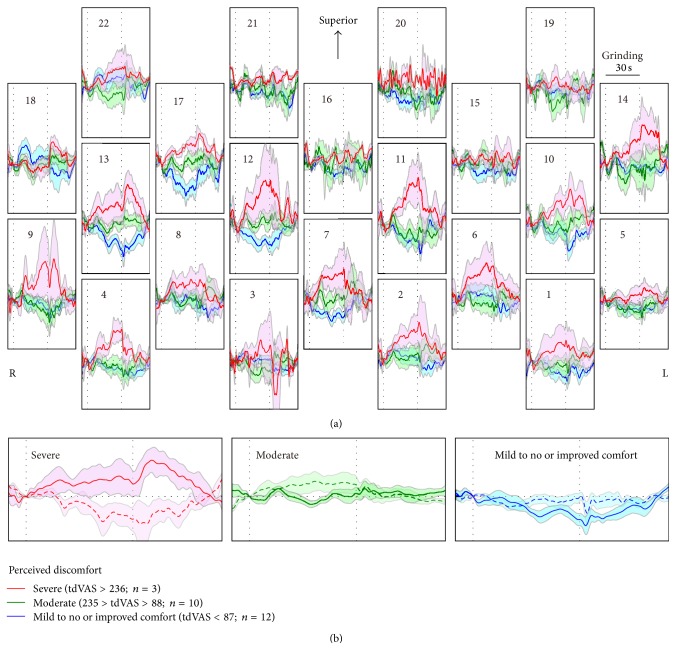
Comparison of the time-course of mean hemodynamic responses in participants with different severities of perceived discomfort. (a) Spatial distribution of the mean ΔOxy-Hb responses over the 22 channels located in the prefrontal area. The number and vertical lines in each subfigure show the corresponding channel number and the timing of grinding behavior. The shaded areas indicate the standard errors of the ΔOxy-Hb responses in the corresponding group. (b) Time-course changes of the mean ΔOxy-Hb (solid line) and the mean ΔdeOxy-Hb (dashed line) responses in participants with different severities of perceived discomfort in a representative channel (13). The shaded areas indicate the standard errors of the ΔOxy-Hb and ΔdeOxy-Hb responses in the corresponding group.

We performed four types of interparticipant analyses to determine the signatures of hemodynamic signals indicating the perceived severity of occlusal discomfort. First, participants were divided into four groups based on the total value of dVAS (tdVAS; Table 1) to examine the signal waveform. Cutoff values were set to mean + 1 standard deviation (1SD), mean, and mean − 1SD of tdVAS. As the number of participants who fell into the group with tdVAS less than mean − 1SD was small (*n* = 2) and the ΔOxy-Hb waveforms from this group were comparable to those from the neighboring group (tdVAS between mean and mean − 1SD; *n* = 10), these two groups were combined together for the schematic representation and statistical comparison of ΔOxy-Hb amplitudes between groups. However, we kept the highest tdVAS group (tdVAS more than mean + 1SD; *n* = 3) apart from the second highest tdVAS group (tdVAS between mean + 1SD and mean; *n* = 10), because the amplitudes of the ΔOxy-Hb responses in several channels demonstrated separated tendencies between the two groups (the statistical significance was confirmed in the analysis described in Section 2.4, also see Figure 2), indicating graded changes in the hemodynamic response depending on the severity of occlusal discomfort.

A two-way analysis of variance (ANOVA) test with repeated measures was adopted to investigate task-dependent ΔOxy-Hb responses and their interaction among groups. We calculated the mean amplitude of ΔOxy-Hb responses during 10–20 s and 20–30 s after starting grinding as the amplitudes of hemodynamic responses during the task period and those during 5–0 s before starting grinding as those during the rest period. The task period was divided into two parts to determine the time-dependent hemodynamic changes during the grinding activity. The rest period and the two task periods were set as the within-subject factor (time) and the three groups were set as the between-subject factor. The difference in the mean amplitudes of ΔOxy-Hb responses among groups was further determined using a post hoc multiple comparison with Bonferroni correction in the channels that showed a statistically significant interaction between the time and group. A Shapiro-Wilk test confirmed the normality of the data used in the analysis. We also processed and analyzed the ΔdeOxy-Hb responses in the same manner as we did with the ΔOxy-Hb responses to confirm the brain-derived temporal patterns of hemodynamic signals. However, we focused on the ΔOxy-Hb responses for the subsequent analysis because of the low signal-to-noise ratio of the ΔdeOxy-Hb responses.

Second, the correlation between the area under the curve of the ΔOxy-Hb responses during the grinding period (AUC) and tdVAS or individual dVAS scores was examined at each channel to further determine fNIRS channels that responded according to the perceived severity of occlusal discomfort. AUC was adopted to determine the cumulative metabolic activity that has been reported to correlate well with the cognitive load in the prefrontal cortex regardless of the individual differences in the time-course shape of the ΔOxy-Hb responses [6]. Either Pearson's correlation coefficient or Spearman's rank correlation coefficient was calculated depending on the result of the Shapiro-Wilk normality test.

Third, AUC and tdVAS were further used in a stepwise multiple-regression analysis to investigate whether a combination of AUC values from selected fNIRS channels would be a reliable predictor of tdVAS, the relative change in perceived discomfort.

Fourth, regional brain activities corresponding to the subtracted Oxy-Hb responses ΔOxy-Hb were identified using statistical parametric mapping (NIRS-SPM; [12]) with a generalized linear model (GLM). The NIRS-SPM converts the individual fNIRS channel positions into the MNI coordinates of the normalized brain and provides the interpolated activity map over the cortical surface from the *T* statistics calculated at discrete fNIRS channels. We took advantage of the interpolated *T*-contrast maps in the normalized brain coordinates to extract the common cortical area among participants that responded to the perceived occlusal discomfort. The participants were divided into two groups who reported severe to moderate discomfort (tdVAS more than mean; *n* = 13) or mild to no discomfort (tdVAS less than mean; *n* = 12) in this NIRS-SPM analysis to increase statistical power. Discrete Cosine Transform-based detrending, which consists of a high-pass filter with a 128 s time constant, was applied to the ΔOxy-Hb responses. The temporal autocorrelation in the ΔOxy-Hb responses was further corrected by a precoloring method [12] using a Gaussian low-pass filter with a full width at half maximum of 1.5 s. Regression models were created using a hemodynamic response function [12] with a duration set at 30 s. The GLM parameters were estimated with the above preprocessed ΔOxy-Hb responses of each participant to obtain an interpolated beta-value map, which indicates the distribution of the task-dependent regional brain activity over the cortical surface covered by the optical probes. We further collected the beta-value maps of the selected participants to estimate the common regional activity using the group analysis function [12]. The extracted task-dependent regional brain activity (*T*-contrast map) was superimposed onto the brain surface image implemented in the NIRS-SPM. The Brodmann areas corresponding to the activated region were further determined using xjview software (http://www.alivelearn.net/xjview8/).

EMG signals from the masseter were sampled at 500 Hz with a band-pass filter of 1.6 s to 1.6 ms time constant (which corresponds to 0.1–100 Hz). The data were imported and further processed in Matlab (Mathworks, Natick, MA, USA). Full-wave rectification and low-pass filtering with a 32 ms time constant (corresponding to 5 Hz) were applied to extract the ridge line of EMG activity. Muscle activities during the rest and grinding periods were extracted and averaged over trials for each participant. We further calculated the increase of the averaged muscle activities related to grinding by dividing those during grinding by those during rest. These grinding-related increases of muscle activities were compared between sessions with and without metal strips using a paired *t*-test.

All data are shown as mean ± standard error unless otherwise stated. We considered *P* values < 0.05 to be statistically significant.

## 3. Results

### 3.1. Grinding with Metal Strips Evoked Varying Intensity of Perceived Discomfort

Table 1 summarizes VAS scores of all participants with their profiles. Experimental occlusal elevation by metal strips led to increased perceived discomfort in most of the participants, although a few of them reported less discomfort or improved comfort while grinding metal strips. Among all participants who underwent the fNIRS or EMG experiments, the chief components of discomfort were uneven contact of the bilateral molar teeth (24.7% of total dVAS scores), unstable IP (19.7%), and “too-high” bite (19.4%), followed by jitteriness of the oral region (16.2%), difficulty in chewing (12.3%), and tooth pain (7.6%). The EMG experiment confirmed that the increase in masseter activity related to grinding was comparable between the two sessions (137 ± 14% and 115 ± 10% in grinding with and without metal strips, resp.; *P* = 0.13; Cohen's *d* = 0.75), indicating that participants could perform grinding with uniform strength regardless of the presence of metal strips.

### 3.2. Prefrontal Oxy-Hemoglobin Responses Indicate Region- and Perceived Discomfort-Specific Patterns

Figure 2(a) shows the complex interactions between perceived discomfort, region of interest, and mean amplitude of ΔOxy-Hb responses. Participants who reported severe discomfort (tdVAS > mean + 1SD, severe group) showed large and bell-shaped ΔOxy-Hb responses in most of the channels. Conversely, participants who reported a tdVAS of less than average, who perceived mild to no discomfort or improved comfort with grinding metal strips (mild to none group), showed suppressed ΔOxy-Hb responses in most of the channels. As expected, the ΔOxy-Hb responses of participants who reported moderate discomfort (moderate group) were mostly located between those from the severe group and the mild to none group, especially in the right hemisphere (channels 12, 13, and 17 in Figure 2(a)). The channels showing increased ΔOxy-Hb response were associated with decreased ΔdeOxy-Hb response (waveforms of a representative channel are shown in Figure 2(b)), which is a typical temporal pattern of cerebral hemodynamics [13].

There was a statistically significant interaction of the mean amplitudes of ΔOxy-Hb responses between time and group in channels 7 (*F*[4,44] = 3.38, *P* = 0.025), 11 (*F*[4,44] = 2.91, *P* = 0.032), 12 (*F*[4,44] = 3.87, *P* = 0.009), 13 (*F*[4,44] = 3.84, *P* = 0.010), and 17 (*F*[4,44] = 4.25, *P* = 0.006), indicating different task-dependent hemodynamic responses among groups. The mean amplitudes of ΔdeOxy-Hb responses accordingly showed a statistically significant interaction between time and group at channel 13 (*F*[4,44] = 5.53, *P* = 0.001). The post hoc multiple comparison with Bonferroni correction showed a statistically significant difference in the mean amplitudes of ΔOxy-Hb responses between the severe and mild to none groups in channels 7 (*t*[13] = 3.01, *P* = 0.023) and 13 (*t*[13] = 3.20, *P* = 0.014) during 10–20 s and in channels 7 (*t*[13] = 3.15, *P* = 0.018), 11 (*t*[13] = 3.04, *P* = 0.020), 12 (*t*[13] = 3.00, *P* = 0.022), 13 (*t*[13] = 3.04, *P* = 0.020), and 17 (*t*[13] = 3.08, *P* = 0.018) during 20–30 s after starting grinding, respectively (*P* < 0.05). The same multiple comparisons also indicated a statistically significant difference in the mean amplitudes of the ΔOxy-Hb and ΔdeOxy-Hb responses between the severe and moderate groups in channels 11 (*t*[11] = 2.70, *P* = 0.040) and 13 (*t*[11] = 2.74, *P* = 0.039) during 20–30 s after starting grinding, respectively. The longer participant continued grinding the more channels showed different mean amplitudes of ΔOxy-Hb response among groups. These results confirmed our hypothesis that prefrontal ΔOxy-Hb responses negatively correlate with perceived occlusal comfort during grinding.

### 3.3. AUC Values of Selected Channels Can Predict Severity of Perceived Discomfort

**Figure 3:**
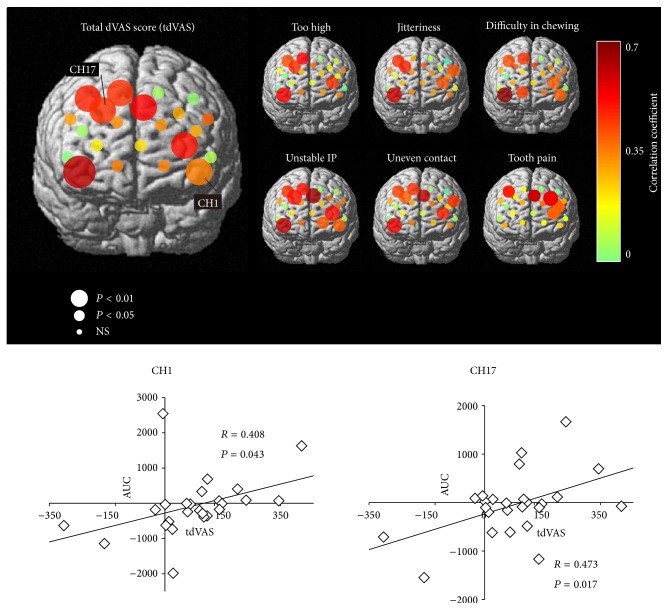
Results of channel-based correlation analysis between AUC and VAS scores. Upper panel: large, medium, and small circle diameters indicate *P* values of less than 0.01, less than 0.05, and equal to or more than 0.05 at the corresponding channel, respectively. The color scale indicates the correlation coefficient. Lower panel: correlated relationship between individual tdVAS scores and AUCs in representative channels. *R* indicates the correlation coefficient. Spearman's rank correlation coefficient was calculated at channels 3, 8, 9, 10, 13, 14, 15, and 16 because data were not normally distributed. Pearson's correlation test coefficient was calculated for the rest of the channels.

The correlation analysis of AUC and VAS scores at each individual channel revealed a significant positive relationship between the severity of perceived discomfort and hemodynamic responses in the prefrontal area (Figure 3). A significant correlation between AUC and tdVAS was found in channels corresponding to the right frontopolar (FPC) and dorsolateral prefrontal cortices (DLPFC), as well as the lateral transitional area of the left FPC and DLPFC. A similar relationship was obtained between AUC and dVAS for all individual discomfort criteria except tooth pain, for which a significant correlation was mostly found in the left FPC and DLPFC. Multiple-regression analysis further indicated that the AUCs of channels 1, 13, and 17 were significant predictors of tdVAS score (coefficient of determination *R* ^2^ = 0.813; *P* < 0.001), obeying the following equation:

(1) tdVAS=0.21AUC(1)+0.132AUC(17)−0.11AUC(13)+77.5,

where AUC (CH) indicates the AUC value at channel CH. The standard partial regression coefficients were *β* = 0.857, 0.631, and −0.474 for channels 1 (*P* < 0.001), 17 (*P* = 0.002), and 13 (*P* = 0.02), respectively.

### 3.4. Occlusal Discomfort Associated with Regional Brain Activity in the Right FPC and DLPFC

**Figure 4:**
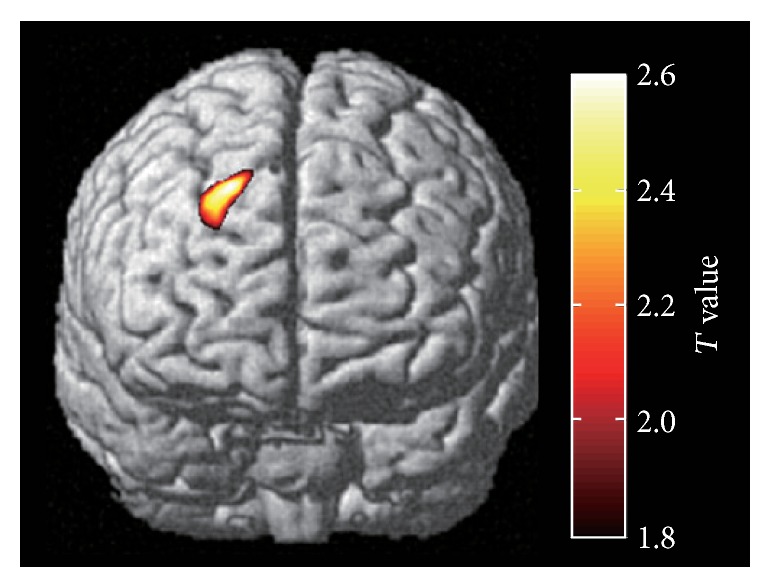
Results of statistical parametrical mapping of 13 participants who reported severe or moderate discomfort while grinding metal strips (*P* < 0.05; uncorrected). The highlighted area corresponds to Brodmann areas 9 and 10 (DLPFC and FPC) in the right hemisphere.

The statistical analysis of ΔOxy-Hb responses from 13 participants who reported severe or moderate discomfort while grinding metal strips indicated localized regional brain activity in the transitional area of the right FPC and DLPFC (Brodmann areas 10 and 9; Figure 4). The localized activity was closely located around channel 17, whose AUC value showed a significant correlation with perceived severity of discomfort in the individual correlation analysis and was a significant explanatory variable in the multiple-regression analysis. We obtained similar right-lateralized regional brain activity in Brodmann areas 9 and 10 when the participants were further divided into two groups depending on their grinding side and analyzed separately (data not shown). No statistically significant regional brain activity was found in the analysis with the subgroup of participants whose tdVAS scores were less than average (*n* = 12; mild to no discomfort or improved comfort in grinding with metal strips).

## 4. Discussion

We used fNIRS to measure prefrontal brain activity accompanying the occlusal discomfort that arose when participants performed grinding with mild occlusal elevation. Canceling out the artifacts related to jaw movement by using differential waveforms between grinding with and without occlusal elevation, we demonstrated a significant relationship between the severity of perceived discomfort and the cumulative hemodynamic responses in the right FPC and DLPFC. These results suggest that fNIRS responses in these prefrontal sites could be good predictors of occlusal discomfort.

Differential hemodynamic responses between two sessions with and without occlusal elevation showed event-related, Gaussian-like responses, confirming that motion artifacts related to the jaw opening and closing were successfully canceled out in most of the channels. The increased ΔOxy-Hb responses associated with decreased ΔdeOxy-Hb responses found in the channels that strongly responded to the grinding behavior in severe perceivers (Figure 2(b)) also show that the differential signals maintain the typical temporal pattern of cerebral hemodynamics. The comparable electromyographic activity between these sessions further suggests that the differential hemodynamic responses represent the sole change in neuronal activity related to somatosensory perception during grinding. Compared with a previously reported noise reduction technique that requires highly complex signal processing [14], our differential method is simple but effective enough to remove the motion- and muscle-originating artifacts in fNIRS signals measured over the forehead region.

Our sensor-level analyses and statistical parametrical mapping consistently showed that the activity in the right FPC and DLPFC correlated well with the severity of occlusal discomfort. Considering that these cortical areas have dense reciprocal connections between the limbic system [15] and respond depending on the cognitive load [6], these results suggest two possible roles of these prefrontal areas, attentional control and self-regulation, in the emotional processing of occlusal discomfort. The decreased hemodynamic activity during the task period in a subgroup of participants who perceived mild to no or improved comfort (Figure 2) may also support this idea because their attention and regulatory activity might be attenuated when they perceive less discomfort when grinding metal strips despite the expectation that they had during the rest period. Using ^15^O-labelled water PET, Kulkarni et al. [16] compared the regional brain activity in two conditions in which participants were instructed to attend to either the location or the unpleasantness of noxious stimuli of the same intensity. While attention to the stimulus location resulted in the activation of the lateral pain system in the primary somatosensory and inferior parietal cortices, attention to the stimulus unpleasantness modified the neuronal responses to enhance the medial pain system, which includes the limbic system such as the amygdala and hypothalamus, the insula, and the FPC.

A PET study on chemically induced heat allodynia [5] further proposed the involvement of the DLPFC in perceiving “unusual” nociceptive stimulation. When compared with equally intense normal heat pain, heat allodynia involved relatively greater recruitment of the medial thalamic pathways, anterior cingulate cortex, orbitofrontal cortex, and DLPFC, which may convey greater affective reactions. Another intervention study using transcranial magnetic stimulation (TMS) demonstrated that induced activation of the right DLPFC improved response time on a cognitive discrimination task, suggesting enhanced attention and top-down control towards the task [17]. These previous findings demonstrate that increased activity in the FPC and DLPFC represents increased attention to afferent sensory information. Our results suggest that participants who reported high severity paid more attention to the elevated occlusion owing to the strong disturbance caused by modified somatosensory sensations from the oral cavity, resulting in enhanced activation in the prefrontal cortex.

Another important cognitive role of these prefrontal areas is to regulate emotional responses. Beauregard et al. [18] reported the activation of the FPC and the concurrent suppression of the limbic areas when participants voluntarily inhibited their emotion while watching highly arousing film clips. Activation of the DLPFC also works to weaken the perceived intensity and unpleasantness of painful thermal stimulation via dampening subcortical regions involved in pain processing pathways [3, 5]. In a repetitive TMS study that recruited 180 healthy volunteers, Graff-Guerrero et al. [4] demonstrated the selective effect of the right DLPFC activation on increasing pain tolerance. The increased FPC/DLPFC activity found in participants with severe perceived discomfort may represent an adaptive response to dampen the unpleasantness owing to the altered occlusion.

At this stage we are not able to conclude whether this increased prefrontal activity during uncomfortable grinding originates from attentional control or from self-regulatory responses to altered somatosensory feedback. Further study using the same experimental design in patients and in healthy volunteers while experimentally manipulating these factors would contribute toward revealing the causal factor for the increased prefrontal activity in occlusal discomfort. If the distraction from the oral sensation during grinding attenuates prefrontal activity in healthy participants and if the correlation between perceived severity of discomfort and prefrontal activity was also preserved in patients, the prefrontal activity would likely represent the intensity of attention to the altered occlusion. In this case, the pathology might arise from an altered or reorganized body image in more primary somatosensory processing systems [19]. As most of the patients have a previous history of newly provided dental treatment such as restorations, dentures, and crowns to trigger their symptoms [1], the excessive attention and reinforcement of the altered occlusion might cause reorganization of the cortical sensory maps to convey unnecessarily detailed sensory information that is not normally available to consciousness [20], resulting in the awareness of occlusal discomfort. However, if prefrontal activity works to regulate emotional responses against the uncomfortable occlusion, the relationship may be distorted in patients owing to the lack of emotional regulatory response. Suppressed basal activity in the prefrontal cortex found in patients with orally localized somatoform pain disorder [2] raises the hypothesis that patients with unexplained occlusal discomfort might have some functional deficits in top-down control that usually ameliorates unpleasant somatosensory input and might therefore show low tolerance even to a slight modification of the occlusion. Concurrent measurement of the somatosensory and prefrontal cortices would further reveal the neuronal relationship between primary and higher centers of sensory perception in the pathophysiology of unexplained occlusal discomfort. Manipulating the emotional regulation in healthy participants while grinding metal strips would also contribute to test this hypothesis.

As a pilot study tackling the quantitative evaluation of the neuronal traits associated with occlusal discomfort, the current results have several limitations. First, we had no “pain only” group with which to compare prefrontal activity to differentiate the effect of pain and discomfort, because we included tooth pain as one of the factors of occlusal discomfort. Further research should be conducted to investigate whether the perception of occlusal discomfort and pain shares analogous neuronal mechanisms or not. Second, care should be taken to interpret the prefrontal activity, because the prefrontal cortex responds to variety of emotional and/or cognitive tasks [21], which suggests the possibility that the activity observed in the current study was a secondary emotional response such as negative emotion not specifically related to the occlusal discomfort. A whole-brain investigation including the limbic system and the somatosensory cortices using functional MRI with the same experimental paradigm would further confirm our current result that increased prefrontal activity reflects the perceived intensity of occlusal discomfort. Third, more investigation is required to confirm the conclusion of this study, because the number of participants who fell into the severe group was small. Further experiments would also benefit from the simultaneous measurement of EMG and fNIRS to allow the grinding force to be consistent between conditions and to avoid muscle artifacts in the fNIRS signals that might remain in the differential signals.

## 5. Conclusions

We demonstrated a positive relationship between the severity of occlusal discomfort and increasing neuronal responses in the right prefrontal cortex. Our active grinding method was capable of capturing the dynamics of the neuronal responses of occlusal discomfort in an event-related manner, which is also applicable to clinical examination. The comparison of the results obtained from symptom-free participants to those with patients would further elucidate the neuronal mechanism of unexplained occlusal discomfort.

## Figures and Tables

**Table 1:** Participant profiles and VAS scores.

Perceived discomfort	Age	Sex	Chewing side	dVAS						Total (tdVAS)
				Too high	Jitteriness	Difficulty in chewing	Unstable IP	Uneven contact	Tooth pain	
(a) NIRS experiment										
Severe	29	F	L	77	81	76	81	84	15	414
	27	M	R	32	66	53	40	100	54	345
	29	F	R	43	64	32	58	35	14	246
Moderate	39	F	L	34	35	36	37	56	22	220
	24	F	R	37	−8	38	46	45	15	173
	28	F	L	32	57	1	14	60	0	164
	34	F	R	24	16	15	37	36	36	164
	24	M	R	2	33	15	14	33	32	129
	26	M	R	31	19	9	33	36	0	128
	26	M	L	28	11	12	22	29	16	118
	25	F	L	33	3	25	26	27	0	114
	22	F	R	19	20	20	34	17	2	112
	33	M	R	26	16	14	29	20	0	105
Mild–none	31	M	R	23	16	21	18	17	−18	77
	31	M	R	23	0	0	24	22	0	69
	27	F	R	14	13	13	13	13	0	66
	24	M	R	4	4	5	5	3	4	25
	26	M	L	11	12	0	0	0	0	23
	26	M	L	6	0	6	0	0	0	12
	24	F	L	0	3	0	0	0	0	3
	29	M	R	−1	−2	−7	5	6	0	1
	24	M	R	0	0	0	0	−6	0	−6
	41	M	R	6	−11	−26	2	1	−1	−29
Comfortable	38	M	R	−33	−31	−29	−32	−41	−17	−183
	36	F	L	−49	−67	−70	−70	−49	−1	−306
Mean	**28.9**			**16.9**	**14.0**	**10.4**	**17.4**	**21.8**	**6.9**	**87.4**
SE	**1.0**			**4.9**	**6.2**	**5.6**	**5.8**	**6.6**	**3.2**	**29.4**

(b) EMG experiment										
	30	M	R	42	26	33	54	58	7	220
	28	F	L	24	24	23	23	24	6	124
	26	M	L	33	5	24	25	24	0	111
	34	F	R	15	3	6	11	16	21	72
	25	M	R	2	36	−1	−2	13	6	54
	34	F	L	5	10	0	3	12	0	30
Mean	**29.5**			**20.2**	**17.3**	**14.2**	**19.0**	**24.5**	**6.7**	**101.8**
SE	**1.6**			**6.4**	**5.4**	**5.9**	**8.2**	**7.0**	**3.1**	**27.6**
